# Combination Image Analysis of Tongue Color and Sublingual Vein Improves the Diagnostic Accuracy of Oketsu (Blood Stasis) in Kampo Medicine

**DOI:** 10.3389/fmed.2021.790542

**Published:** 2022-03-03

**Authors:** Akira Morita, Aya Murakami, Keigo Noguchi, Yuki Watanabe, Toshiya Nakaguchi, Sadayuki Ochi, Kazuho Okudaira, Yoshiro Hirasaki, Takao Namiki

**Affiliations:** ^1^Department of Japanese-Oriental (Kampo) Medicine, Graduate School of Medicine, Chiba University, Chiba, Japan; ^2^Faculty of Pharmacy, Center for Pharmaceutical Education, Yokohama University of Pharmacy, Yokohama, Japan; ^3^Department of Medical Engineering, Graduate School of Science and Engineering, Chiba University, Chiba, Japan; ^4^Department of Research and Development, Center for Frontier Medical Engineering, Chiba University, Chiba, Japan

**Keywords:** Kampo medicine, tongue diagnosis, sublingual vein, tongue color, tongue image analyzing system, Oketsu (blood stasis)

## Abstract

**Aim:**

In tongue diagnosis, a dark purple tongue and enlarged sublingual vein are important findings of Oketsu (blood stasis). However, the association between the tongue color and the sublingual vein has not been reported. This study investigated the association between the tongue color values and the sublingual vein width using tongue image analyzing system (TIAS) for the objective assessment of blood stasis.

**Methods:**

A total of 38 patients (age 68.7 ± 11.3 years, 14 men and 24 women) who visited the Department of Kampo Medicine at Chiba University Hospital were included. Physical findings, blood test results, blood stasis score from medical records, and tongue images obtained with TIAS were analyzed. The patients were classified into two groups: patients with a sublingual vein width of ≤2.5 mm (20 patients) and those with a width of >2.5 mm (18 patients). The physical findings and the blood test results of the two groups were analyzed by Wilcoxon's rank-sum test or χ^2^-test, whereas logistic regression analysis was used to determine the association between the tongue color values and sublingual vein width. Receiver operating characteristic (ROC) analysis was used to differentiate blood stasis.

**Results:**

The color values significantly related to the sublingual vein width (mm) were the P1-L^*^ and P4-L^*^ (darkness of the tongue edge and tongue apex) and the P1-b^*^ and P2-b^*^ (blueness of the tongue edge and tongue posterior). The area under the curve was greater for the combination of the tongue color values and the sublingual vein width than that for either of them.

**Conclusion:**

This study demonstrated an objective evaluation of blood stasis in the tongue of patients with dark-blue discoloration and an enlarged sublingual vein. In addition, the combination of the tongue color and the sublingual vein is expected to facilitate a more reliable diagnosis of blood stasis.

## Introduction

Tongue diagnosis in Kampo medicine based on its color and shape is useful for understanding the general condition of patients. Western medicine research has also reported that the moisture, color tone, and shape of the tongue reflect the body's water metabolism and health condition (1, 2). However, the assessment of tongue color is considered to be easily influenced by environmental conditions, such as light source and room temperature, as well as the subjective judgment of the physician based on knowledge and experience. Therefore, standardization of tongue diagnosis and better support for Kampo medicine education are required (3). In recent years, we have developed a tongue image analyzing system (TIAS) to support tongue diagnosis (4, 5). Tongue image analyzing system uses an integrating sphere and diffuse light illumination to maintain a constant light intensity and provide a stable image of the tongue without gloss on the surface while under stable conditions unaffected by environmental factors, such as light source, dryness, and room temperature. From the captured images, it is possible to quantify the tongue color using the sublingual vein and the L^*^a^*^b^*^ color system, which is a color space data set developed by the Commission Internationale de l'Éclairage. This is equally useful for training and the objective evaluation of tongue findings, which are important in Kampo medicine. We have previously reported the usefulness of TIAS beyond the field of Kampo medicine (6) and highlighted its potential usefulness as a non-invasive screening tool for gastric cancer (7).

Oketsu (blood stasis) is considered to be caused by the stagnation of blood, and is one of the key pathological concepts in Kampo medicine (8). However, assessment of blood stasis may be complex from the perspective of Western medicine. In recent years, there have been attempts to quantify the findings of blood stasis. It has been reported that blood rheological abnormalities, such as blood viscosity, erythrocyte aggregation, and fibrinogen are observed in blood stasis (9, 10), and that blood stasis is associated with blood fluidity (11) and coronary artery disease (12), which are regarded as microcirculatory disorders in Western medicine (13).

Since ancient times, tongue diagnosis has been used to understand the pathogenesis of blood stasis. A dark purple tongue along with the color tone, curvature, and distension of the sublingual vein have been regarded as signs of blood stasis (14). There was a positive correlation between the enlarged sublingual vein and varicose veins in the lower extremities (15); moreover, the color of the sublingual vein strongly correlated with the pathology of blood stasis and tongue color (16, 17). We previously reported that the sublingual vein width tended to widen in patients with blood stasis based on TIAS (6). However, there are no reports of detecting blood stasis based on the combination of the tongue color and the sublingual vein. Therefore, in this study, we objectively evaluated the association between the tongue color and sublingual vein in patients with blood stasis using TIAS. We determined the tongue color values and sublingual vein width in patients who underwent tongue imaging using TIAS and assessed the objective indicator for differentiating blood stasis.

## Methods

### Participants

We conducted a retrospective study of 38 patients (14 men and 24 women) aged 20 years or older who visited the Department of Kampo Medicine at Chiba University Hospital from May to August 2017. These patients provided consent for their tongue images to be taken using TIAS. The patients were fully informed about the use of the TIAS tongue images, and the test results for research purposes at their first visit were obtained. This study was approved by the Ethics Committee of Chiba University (Approval No.: 812) and was conducted according to the principles of the Declaration of Helsinki. Efforts were made to prevent personal identification based on patient information.

### Study Design

#### Tongue Image Analyzing System

**Figure 1:**
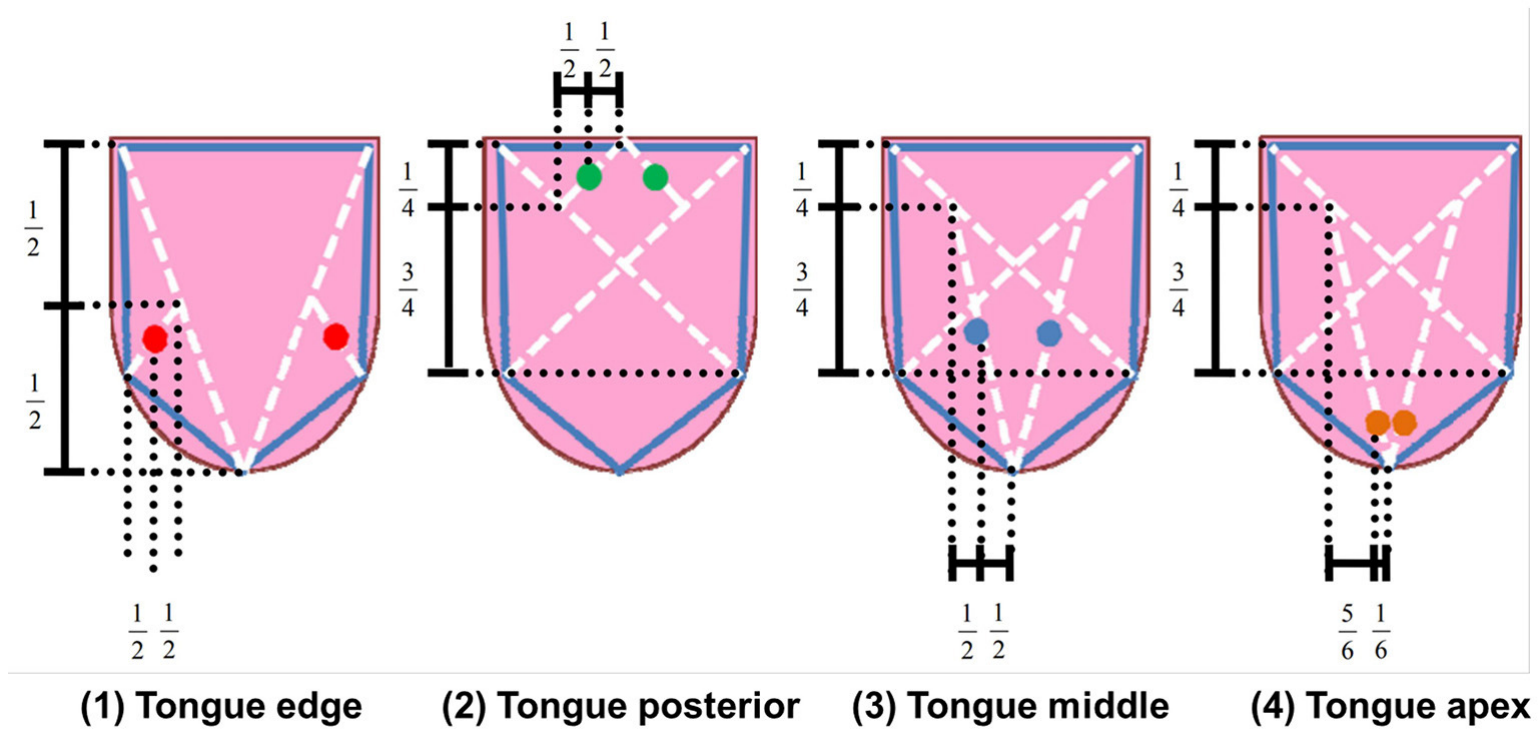
Definition of the four measurement points of the tongue color values.

The blood test results and physical findings of 38 patients were obtained from their medical records, whereas tongue images were obtained using TIAS. To determine the tongue color, we converted the color values from RGB to L^*^a^*^b^*^, using the tongue images (7). The L^*^a^*^b^*^ color space is a device-independent color space comprising L^*^ for lightness (brightness to darkness), a^*^ for chromaticity (redness [+a] to greenness [–a]), and b^*^ for chromaticity (yellowness [+b] to blueness [–b]). Four measurement points were used: P1 (tongue edge), P2 (tongue posterior), P3 (tongue middle), and P4 (tongue apex). The ratios used to determine the four measurement points by specifying five spots on the tongue contour are shown in Figure 1. The four measurement points were calculated as the average of the tongue color values of two circles on the left and the right, which had a diameter of 5 mm. P1 and P4 were assessed for the color of the tongue texture. P2 and P3 were assessed for the color of the tongue texture and that of the tongue fur.

Based on the tongue images taken by TIAS, the sublingual vein width was determined using an image processing software (Image J). The measurement was taken from close to the tongue root to the point where the diameter of either the right or left blood vessel of the vein, not including a vascular bifurcation, was maximal (6).

#### Classification Based on the Sublingual Vein Width

We investigated the relationship between the physical findings, blood test results, and tongue color values of the patients due to differences in the sublingual vein width. The mean sublingual vein width of the 38 patients was 2.5 mm, which was then divided into ≤ 2.5 mm group (*n* = 20) and >2.5 mm group (*n* = 18). The strength of the association between the physical findings, blood test results, and tongue color values were analyzed in the two groups.

#### Indicator of Blood Stasis

A total of 38 patients were classified into the non-blood stasis group (*n* = 18) and the blood stasis syndrome group (*n* = 20) with the blood stasis score, which could assess objectively blood stasis (18). Cutoff values were calculated using receiver operating characteristic (ROC) analysis. The blood stasis score contains 17 evaluation items, with total scores ranging from 0 to 90 for males and 0 to 101 for females. Regardless of sex, patients with a total score <21 were classified in the non-blood stasis group, and those with a total score ≥21 were classified in the blood stasis syndrome group (Table 1). Each finding required for the score was determined by multiple experts with ≥10 years of experience in clinical practice (3), particularly in Kampo medicine.

**Table 1:** Diagnostic criteria for blood stasis.

**Symptom**	**Score**	
	**Male**	**Female**
Dark-rimmed eyes	10	10
Areas of dark-pigmentation of facial skin	2	2
Rough skin	2	5
Livid lips	2	2
Livid gingiva	10	5
Livid tongue	10	10
Telangiectasis/Vascular spiders	5	5
Subcutaneous hemorrhage	2	10
Palmar erythema	2	5
Resistance and tenderness on pressure of the left para-umbilical region	5	5
Resistance and tenderness on pressure of the right para-umbilical region	10	10
Resistance and tenderness on pressure of the umbilical region	5	5
Resistance and/or tenderness on pressure of the ileocecal region	5	2
Resistance and/or tenderness on pressure of the sigmoidal region	5	5
Resistance and/or tenderness on pressure of the subcostal region	5	5
Hemorrhoids	10	5
Dysmenorrhea	–	10

*Regardless of sex, patients with total scores <21 were classified into the non-blood stasis, and those with total scores ≥21 were classified into the blood stasis syndrome. Mild symptoms are designated by half points*.

## Statistical Analysis

All the data are summarized as mean ± standard deviation. Wilcoxon's rank-sum test or χ^2^-test was used to compare the physical findings and blood test results between the two groups based on the sublingual vein width. Logistic regression analysis was used to evaluate the association of tongue color values, while ROC analysis was used to differentiate blood stasis after we classified the blood stasis scores into the non-blood stasis and blood stasis syndrome group. All statistical analyses were performed using JMP version 14 (SAS Institute Japan Ltd.), and *p* < 0.05 was considered statistically significant.

## Results

### Classification Based on the Sublingual Vein Width

This study included several patients with glucose intolerance; therefore, fasting plasma glucose and hemoglobin A1c (%) were slightly higher than normal in both groups. The items that were significantly higher in the >2.5 mm group were blood stasis score and LDL-cho level (Table 2).

**Table 2:** Characteristics of patients.

**Sublingual vein width (mm)**	**≤2.5 (***n*** = 20)**	**>2.5 (***n*** = 18)**	***p*** **-Value**
**Physical findings**			
Sex (M/F)	6/14	8/10	0.501
Age (year)	64.9, 13.9	73.1, 8.9	0.104
BMI (kg/m^2^)	22.7, 4.1	24.6, 3.5	0.133
Blood stasis score	22.1, 11.8	33.9, 14.4	0.047*
**Blood test results**			
TG (mg/dL)	118.8, 61.2	119.5, 67.0	0.589
HDL-cholesterol (mg/dl)	62.9, 20.8	58.7, 13.1	0.619
LDL-cholesterol (mg/dl)	115.8, 41.7	126.2, 31.1	0.049*
HbA1c (NGSP, %)	6.4, 1.0	6.4, 0.8	0.965
FPG (mg/dl)	120.1, 34.3	120.3, 24.2	0.770

*BMI, body mass index; TG, triglyceride; HDL, high-density lipoprotein; LDL, low-density lipoprotein; HbA1c, hemoglobin A1c; FPG, fasting plasma glucose. Data are presented as the mean ± SD*.

* *p > 0.05, Wilcoxon rank-sum test*.

**Figure 2:**
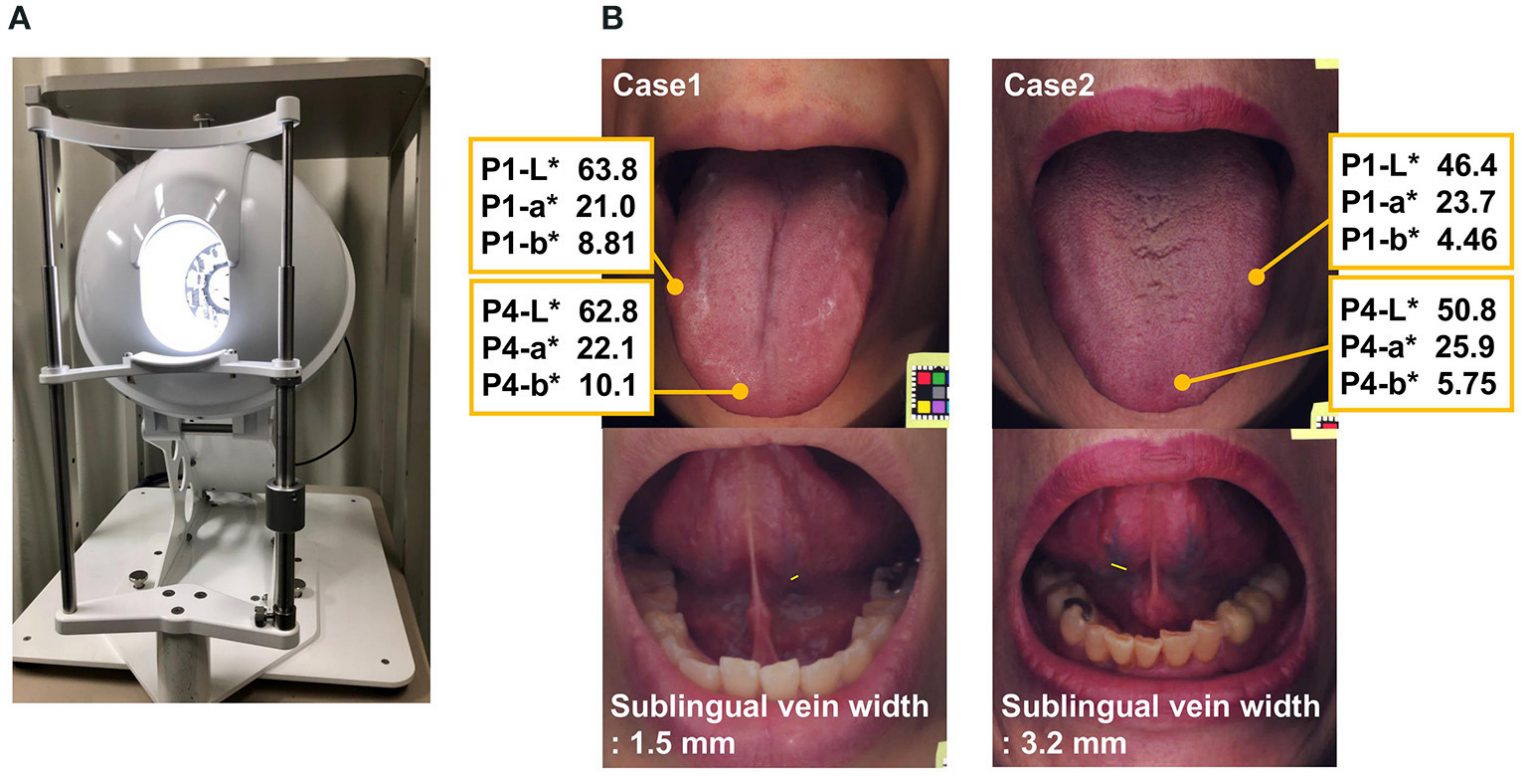
**(A)** Tongue Image Analyzing System: TIAS. **(B)** Tongue images of two clinical cases. Top: The tongue color values. Bottom: The sublingual vein width. Case 1. Blood stasis score 20, Case 2. Blood stasis score 39. P1 (tongue edge), P4 (tongue apex). L* for lightness (brightness to darkness), a* for chromaticity (redness [+a] to greenness [–a]), and b* chromaticity (yellowness [+b] to blueness [–b]).

The L^*^ values (darkness) of P1 and P4 as well as the b^*^ values (blueness) of P1 and P2 were significantly associated with the sublingual vein width. Moreover, the L^*^ values (darkness) of P3 tended to influence the sublingual vein width (Table 3). Tongue image analyzing system and examples of tongue images are shown in Figure 2.

**Table 3:** Association between the tongue color values and sublingual vein width.

	**Sublingual vein width (mm)**		**OR**	**95% CI**	***p*** **-Value**
	**≤2.5 (***n*** = 20)**	**>2.5 (***n*** = 18)**			
P1-L*	53.93, 4.28	49.32, 5.83	1.22	1.04–1.42	0.004*
P2-L*	43.04, 16.94	37.98, 15.26	1.02	0.98–1.06	0.405
P3-L*	58.16, 5.34	55.63, 5.68	1.12	0.98–1.28	0.069
P4-L*	53.14, 4.64	49.30, 5.83	1.18	1.02–1.37	0.014*
P1-a*	25.03, 4.30	24.14, 3.90	1.03	0.88–1.21	0.687
P2-a*	20.81, 6.92	20.77, 6.58	1.00	0.91–1.10	0.959
P3-a*	23.47, 5.07	21.14, 5.61	1.08	0.95–1.24	0.239
P4-a*	29.75, 5.70	28.49, 5.34	1.03	0.91–1.16	0.635
P1-b*	6.43, 2.51	4.83, 1.85	1.44	1.03–2.03	0.019*
P2-b*	7.98, 3.43	5.27, 3.58	1.30	1.04–1.63	0.009*
P3-b*	6.13, 2.40	5.83, 2.78	1.09	0.84–1.41	0.499
P4-b*	6.97, 2.51	5.92, 2.05	1.19	0.89–1.59	0.224

P1 (tongue edge), P2 (tongue posterior), P3 (tongue middle), P4 (tongue apex). L* for lightness (brightness to darkness), a* for chromaticity (redness [+a] to greenness [–a], and b* chromaticity) (yellowness [+b] to blueness [–b]). Data are presented as the mean ± SD.

* *p < 0.05, logistic regression analysis*.

### Indicator of Blood Stasis

**Figure 3:**
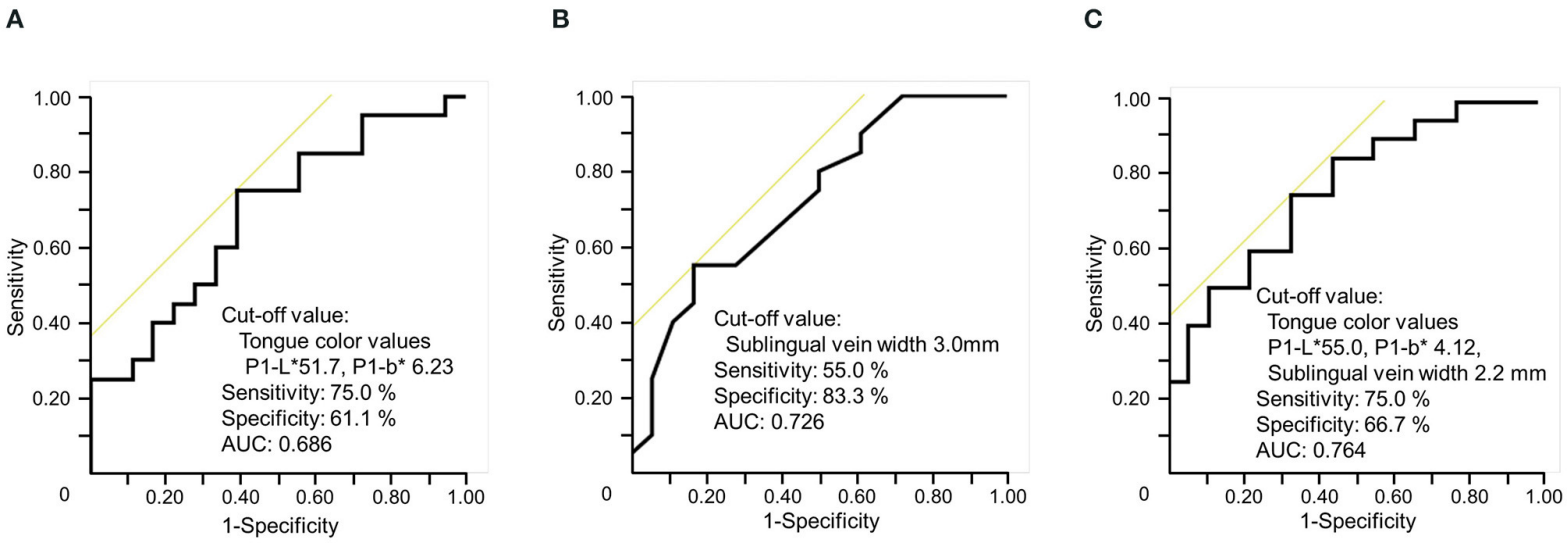
Indicator to differentiate blood stasis by ROC analysis. **(A)** Only the tongue color values (P1-L*, P1-b*). **(B)** Only the sublingual vein width. **(C)** Both the tongue color values (P1-L*, P1-b*) and the sublingual width P1 (tongue edge). L* for lightness (brightness to darkness), b* chromaticity (yellowness [+b] to blueness [–b]).

A total of the 38 patients, 18 and 20 were included in the non-blood stasis group and blood stasis syndrome group, respectively, based on their blood stasis score. Receiver operating characteristic analysis was used to search for indicators to differentiate blood stasis. The highest area under the curve was obtained by combining the tongue color values (P1-L^*^, P1-b^*^) and sublingual vein width (Figure 3) compared to AUCs of these two.

## Discussion

To the best of our knowledge, this is the first study to report on the relationship between tongue color values and sublingual vein width using objective methods among patients with blood stasis. Factors affecting sublingual vein width included P1-L^*^, P-3L^*^, and P-4L^*^, indicating darkness of the tongue, as well as P1-b^*^ and P2-b^*^, indicating blueness of the tongue. Our findings provide objective evidence for the empirical subjective methods, as a dark purple tongue and sublingual vein vasodilation are signs of blood stasis (14). In addition, this study shows the importance of detecting blood stasis at the edge of the tongue, which is the least affected by tongue fur.

P2-L^*^ values were lower (darker) than those of P1-L^*^, P3-L^*^, and P4-L^*^. The reason for this is that sticking out the tongue until the posterior portion can be seen affects the original color and shape of the tongue. In this study, patients were instructed to open their mouths and put out their tongue without straining; therefore, the lower value of P2-L^*^ relative to the others can be considered clinically significant. There were no significant differences in the b^*^ values among the four points (P1, P2, P3, P4), while the a^*^ values of the two groups tended to be higher (redder) in P4 than in P1, P2, and P3. The edge and apex of the tongue can be easily assessed to determine the tongue color, since they are less susceptible to tongue fur. However, redness of the tongue apex is one of the findings that may indicate heart and/or lung heat in Kampo medicine, which may be associated with anxiety and/or common cold. Consequently, the a^*^ values can be high because of these factors; therefore, to obtain them accurately, it would be better to assess the edge and apex of the tongue simultaneously.

Furthermore, the combination of the tongue color values and sublingual vein width provided high diagnostic accuracy to differentiate blood stasis. The cutoff values (P1-L^*^: ≤ 55.0, P1-b^*^: ≤ 4.12) are considered to be useful for the early detection of blood stasis (sublingual vein width: ≥2.2 mm). The edge of the tongue is narrow, and thus may be difficult to assess due to tongue fur. In addition, we previously reported that approximately 25% of patients are unable to turn their tongues (6), thus making it difficult to determine the sublingual vein width. Therefore, it can be assumed that a diagnostic accuracy may be deemed more reliable if it was based on both the tongue color values and sublingual vein width.

The blood stasis score is a diagnostic criterion developed based on scientific interventions (three analytical methods: multiple regression analysis, discriminant analysis, and principal component analysis) with the descriptions and experiences of predecessors established in the long history. The dark purple tongue is shown as livid tongue in the blood stasis score. Additionally, the relationship between blood stasis and tongue color has already been established. In this study, tongue color was objectly evaluated as a clinically acceptable indicator.

The quantification of tongue findings has been attracting attention globally (19–22), and if the accumulation of data for TIAS is encouraged in the future, it will facilitate better understanding of the pathological conditions and tongue diagnosis. This would provide support to medical personnel who are inexperienced in tongue diagnosis.

Our study had some limitations. First, we calculated the vessel diameter in the sublingual vein using analysis software, although the possibility of minimal errors caused by manual implementation of the methods cannot be ruled out. In the present study, measurement rules were established in advance for all the analyses, and only one trained person was allowed to perform such; therefore, the error was extremely small upon excluding individual differences. However, further improvement of the device is necessary to capture the veins at the back of the tongue more clearly and to improve accuracy.

Second, several patients had impaired glucose tolerance because we included consecutive patients who visited to our department during the study period. We have not been able to examine whether similar results can be obtained in patients without impaired glucose tolerance or those of other ages or with other diseases. In addition, we were unable to thoroughly investigate other factors that affect the sublingual vein width. In the future, we plan to expand the number of patients and conduct a multivariate analysis including various factors.

Despite the above limitations, the L^*^ and b^*^ values in the edge of the tongue were quantified for the first time using an objective method, which are important for differentiating blood stasis and TIAS. Moreover, it is considered a useful aid in tongue diagnosis. We hope that this study will contribute to further understanding and development of Kampo medicine.

## Data Availability Statement

The original contributions presented in the study are included in the article/Supplementary Material, further inquiries can be directed to the corresponding author/s.

## Ethics Statement

The studies involving human participants were reviewed and approved by the Ethics Committee of Chiba University. The patients/participants provided their written informed consent to participate in this study. Written informed consent was obtained from the individual(s) for the publication of any potentially identifiable images or data included in this article.

## Author Contributions

AMo and YW carried out the experiment. AMo and AMu wrote the manuscript with support from SO, KO, YH, and TN. AMu and KN analyzed the data with support from TN. AMo and TN conceived the original idea. TN supervised the project. All authors discussed the results and contributed to the final manuscript.

## Funding

The authors are extremely grateful to the Japan Agency for Medical Research and Development (AMED) for providing funding (Grant No: JP211k0310078) in this study. The authors are also extremely grateful to Takano Co., Ltd. for providing TIAS through AMED funding and providing continued maintenance through the same funding. Takano Co., Ltd. was not involved in the study design, collection, analysis, interpretation of data, the writing of this article or the decision to submit it for publication.

## Conflict of Interest

The authors declare that the research was conducted in the absence of any commercial or financial relationships that could be construed as a potential conflict of interest.

## Publisher's Note

All claims expressed in this article are solely those of the authors and do not necessarily represent those of their affiliated organizations, or those of the publisher, the editors and the reviewers. Any product that may be evaluated in this article, or claim that may be made by its manufacturer, is not guaranteed or endorsed by the publisher.
